# Unexpected monophyletic origin of *Ephoron shigae* unisexual reproduction strains and their rapid expansion across Japan

**DOI:** 10.1098/rsos.150072

**Published:** 2015-06-09

**Authors:** K. Sekiné, F. Hayashi, K. Tojo

**Affiliations:** 1 Biological Science in Mountainous Area, Interdisciplinary Graduate School of Science and Technology, Shinshu University, Asahi 3-1-1, Matsumoto, Nagano 390-8621, Japan; 2 Department of Biology, Faculty of Science, Shinshu University, Asahi 3-1-1, Matsumoto, Nagano 390-8621, Japan; 3 Institute of Mountain Science, Shinshu University, Asahi 3-1-1, Matsumoto, Nagano 390-8621, Japan; 4 Division of Insect Sciences, National Institute of Agrobiological Sciences, 1-2, Owashi, Tsukuba, Ibaraki 305-8634, Japan; 5 Department of Biology, Tokyo Metropolitan University, Minamiosawa 1-1, Hachioji, Tokyo 192-0397, Japan

**Keywords:** automixis, geographical parthenogenesis, phylogeography, thelytoky

## Abstract

The burrowing polymitarcyid mayfly *Ephoron shigae* is distributed across Japan, Korea, northeast China and far east Russia. Some populations are bisexual, and others are unisexual, i.e. geographically parthenogenetic throughout Japan. In general, parthenogenetic organisms are often found in harsh environments, such as at high latitudes and altitudes, in xeric as opposed to mesic conditions, in isolated habitats such as islands and island-like areas, and at the peripheral regions of the taxon's range. In *E. shigae*, however, the distributions of bisexual and unisexual populations overlap broadly in their respective geographical ranges. In the analysis of mitochondrial 16S rRNA and COI, we revealed that unisexual populations were of monophyletic origin and recently differentiated somewhere in western Japan. In the nuclear DNA EFI-*α* analysis, parthenogenetic strains had two genotypes, i.e. the heterozygous genotype of E1/E3 and the homozygous genotype of E1/E1 or E3/E3, while specimens of bisexual lineage had 20 genotypes. These results are consistent with an automixis mode of reproduction for the parthenogenetic strains, and also support the monophyletic origin of the parthenogenetic strains. Furthermore, there would be no gene flow between the specimens of the bisexual lineage and those of the parthenogenetic strain.

## 1. Introduction

Most metazoans perpetuate generations via sexual reproduction [1]. The origin and evolution of sexual reproduction has been considered an enigma, and discussed for a long time within evolutional biology [2,3]. It is a paradox that bisexual reproduction, which is a sexually reproductive system relying on a male and a female, is the general mode of reproduction for metazoans, especially when considering that bisexual reproduction has a twofold cost compared with unisexual organisms with the ability to generate all female offspring (e.g. [2]).

Sexual reproduction has occurred widely in multicellular organisms, while some organisms have secondarily lost the capacity for sexual reproduction, while reproduction has been reported in 19 of 34 phyla in the animal kingdom, i.e. Animalia [4]. Parthenogenetic organisms have been recognized as being efficient subjects for the study of evolution and correspondingly the significance of sex (cf. [5]). One particularly remarkable area of study focuses on geographical parthenogenesis. That is, the geographically distinct distribution of closely related bisexual and parthenogenetic organisms [6]. In general, it is well documented that parthenogenetic reproduction is most often found in harsh environmental habitats, such as at higher latitudes and altitudes, mountainous regions, some isolated conditions such as islands or in island-like habitats, in xeric as opposed to mesic environments, and in environments variously classified as periphery, extreme, stressful, transient or disturbed [1,7–11].

**Figure 1.**
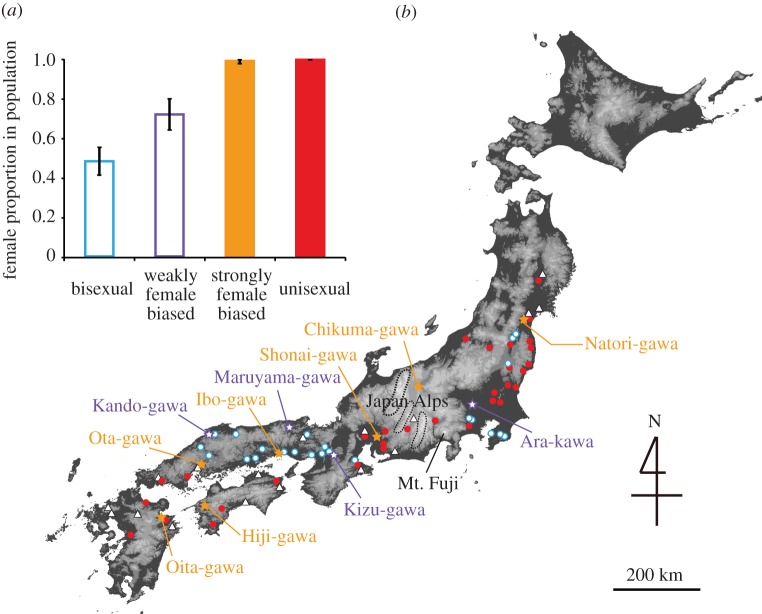
(*a*) Sex ratios of populations in *Ephoron shigae*. Bar graphs and error bars indicate averages and standard deviations of nymphal sex ratios in bisexual (*n*=12), weakly female biased (*n*=4), strongly female biased (*n*=5) and unisexual populations (*n*=23). (*b*) Distribution of *E. shigae* in Japan. Colours of population types are the same as bar graphs (*a*). Blue open circles indicate bisexual populations, red solid circles unisexual, purple open stars weakly female biased, and orange solid stars strongly female biased.

The burrowing polymitarcyid mayfly, *Ephoron shigae* (Takahashi) is distributed widely within far east Asia (figure 1) [12–14] and is also a geographically parthenogenetic species [15–18]. In *E. shigae*, however, the distributions of bisexual and unisexual populations overlap broadly in their respective geographical ranges throughout Japan (Honshu, Shikoku and Kyushu). Obligatory parthenogenesis (i.e. parthenogenesis is the normal mode of reproduction) is observed within unisexual populations [16]. Furthermore, all the individuals that reproduced by parthenogenesis were diploid females (2*n*=12; sex-chromosome type was XX), indicating the process of thelytokous parthenogenesis [17,18]. In our previous study, we revealed the necessary completion of the following two processes in order to achieve a successful return to the diploid phase for parthenogenetic reproduction [18]: (i) complete meiosis occurs in oogenesis; and (ii) following meiosis, the egg nucleus (i.e. female pronucleus) and its sister nucleus of the second polar body fuse together automicticly.

*Ephoron shigae*, therefore, is a uniquely well-suited model to study the differentiation of unisexual and bisexual populations, the establishment of parthenogenesis, and the dispersal of parthenogenetic individuals. An important step in understanding patterns of evolution in parthenogens is to distinguish the cases of single versus multiple origin from a bisexual ancestor [4].

In this study, we examined sex ratio of populations by our field study, and literature survey and classified population types, i.e. unisexual or bisexual populations. Subsequently, we investigated the nature of the origin of the parthenogenetic strains studied using molecular phylogenetic analyses in order to elucidate whether each parthenogenetic strain is of singular or multiple origin. In addition, we discussed dispersal ability and reproduction mode of parthenogenetic strain and evolutionary process of geographical parthenogenetic populations distributed mosaically.

## 2. Material and methods

### 2.1 Distribution and sex ratio of *Ephoron shigae* populations

We gathered data on the distribution and sex ratio of the mayfly *E. shigae* based on our own field research and on surveys of newly published literature and records. In our study from 1999 to 2013, the sex ratio was examined with the ultimate instar nymphs collected on a scale of approximately 100 individuals in each population by qualitative sampling using a D-frame net (1.0×1.0 mm mesh). In the field, we fixed samples of nymphs in 99.5–100% ethanol, and then observed the presence or absence of the viewable primordia of forceps and penes (i.e. determination of sex), under a microscope in a laboratory.

### 2.2 Sample collection and DNA extraction

Four hundred and twenty-two specimens of *E. shigae* were collected from 1999 to 2013 from 40 populations covering most of its distributional range in Japan. Most specimens of nymphs, subimagos and imagos were fixed in 99.5–100% ethanol in the field, and several specimens were fixed in 70% ethanol and then transferred to 99.5–100% ethanol for long-term storage.

Total genomic DNA was extracted from ethanol-preserved tissues of specimens and purified using a DNeasy-Tissue-Kit (QIAGEN, Hilden, Germany) and the standard cetyltrimethylammonium bromide protocol [19].

### 2.3 DNA sequencing

The mitochondrial 16S rRNA and COI genes and the nuclear EFI-*α* genes were amplified by the polymerase chain reaction (PCR) method using the primer sets (16S rRNA: 5′-TTACGCTGTTATCCCTAA-3′ and 5′-CGCCTGTTTATCAAAAACAT-3′[20]; COI: 5′-GGTCAACAAATCATAAAGATATTGG-3′ and 5′-TAAACTTCAGGGTGACCAAAAAATCA-3′ or 5′-CCCGGTAAAATTAAAATATAAACTTC-3′ [21,22]; EFI-*α*: 5′-CCTGGGTGTTGGACAAGCTCAAG-3′ and 5′-AAGACGCAGGGGCTTCTCTG-3′). PCR products were purified using the Microcon-Kit (MILLIPORE, Massachusetts, USA) or the ExoSap-IT (GE Healthcare UK, Buckinghamshire, UK). Purified DNA fragments were sequenced directly by an automated method using the DYEnamic-ET-Terminator Cycle-Sequencing Kit (GE Healthcare UK) and BigDye Terminator v. 1.1 Cycle-Sequencing Kit (Applied Biosystems, California, USA) on an automated sequencer (ABI PRISM 377 and ABI 3130*xl* DNA Analyzer (Perkin Elmer/Applied Biosystems)).

### 2.4 Sequencing alignment and phylogenetic analysis

We aligned all sequences using CLC Workbench software (CLC bio, Aarhus, Denmark), and cross-checked them by a Clustal W [23] algorithm implemented in MEGA5.10 [24]. The PHASE algorithm [25,26], as implemented in DnaSP v. 5 [27], was used to reconstruct putative alleles of the nuclear EFI-*α* gene. 16S rRNA and COI haplotypes and EFI-*α* genotypes have been submitted to the DNA databank of Japan (DDBJ database) with accession numbers AB874274–AB874406. As outgroups, we used 16S rRNA (accession numbers: AB711583 and AB711590) and COI sequence (AB711757 and AB711764) of *E. shigae* in Korea [13,14]. Phylogenetic analyses were performed by Bayesian inference (BI) methods in MrBayes v. 3.2.5 [28–30]. The optimal models of nucleotide substitution and the schemes were selected by the Akaike information criteria in Kakusan4 [31,32] and the marginal likelihood estimations using the stepping-stone and the harmonic means. The best-fit substitution models were chosen as HKY 85+G+I (16S rRNA), GTR (COI 1st and 2nd codons) and GTR+G+I (COI 3rd codon) models for the proportional and codon proportional model. BI analyses were performed by setting the number of Markov-chain Monte Carlo (MCMC) generations at three million, setting the sampling frequency at 100 and calculating a consensus topology after discarding the first 25% samples. Statistical support for the resultant BI trees was determined with Bayesian posterior probabilities (BPP).

In the nuclear EFI-*α* gene, a statistical parsimony tree (network tree) was calculated using TCS v. 1.21 [33]. Gaps were not evaluated as characters in the analysis.

### 2.5 Population genetic analysis

Divergence among the bisexual populations was assessed by an analysis of molecular variance (AMOVA) based on COI and EFI-*α*, using Arlequin v. 3.01 [34]. The statistical significance of each of the variance components of the AMOVA and the paired comparisons was determined by non-parametric procedures using 1000 random permutations. We also performed Mantel tests on the pairwise genetic and geographical distance matrices, using Arlequin v. 3.01 [34].

We also used COI data to infer patterns of demographic history in sexual *E. shigae*. First, two tests based on the distribution of segregating sites (Tajima's D) and on the haplotype distribution (Fu's FS) were applied to the data. Significance of D and FS statistics was tested by generating 10 000 coalescent simulations in Arlequin v. 3.01 [34]. To further investigate the possibility of demographic expansions, we plotted the distribution of pairwise sequence differences (mismatch distribution) for COI haplotypes. The goodness of fit between the observed and expected distributions of a sudden expansion model was calculated using the sum of square deviations (SSD). The significance of Harpending's raggedness index (RI) was calculated using a null distribution simulated from 1000 permutations in Arlequin.

The past population dynamics of *E. shigae* through time were further estimated using a coalescent-based Bayesian skyline plot without the assumption of particular demographic models [35]. The posterior probability distribution of effective population sizes (Nes) and time to the most recent common ancestor (Tmrca) were estimated in BEAST v. 1.7.4 [36], using the best-fitted model of nucleotide substitutions and their parameter values as priors. The nucleotide substitution rate of COI was set to the average value commonly found in arthropods (1.77% divergence lineages^−1^ Myr^−1^; [37] and 0.75%; [38,39]) with a strict molecular clock. MCMC samplings were run for 1.5×10^7^ generations with parameters sampled for every 1×10^3^ generation. The initial 10% of the run was discarded as burn-in. Each analysis was repeated multiple times to optimize the scale factors until no suggestion message appeared on the log file. The effective sample size (ESS) for the posterior distribution of estimated parameter values was determined using Tracer v. 1.5 [40].

## 3. Results

### 3.1 Sex ratios of local populations

We investigated the sex ratios of forty-six populations in Japan by means of our own fieldwork and based on previously published studies (figure 1; electronic supplementary material, table S1). Thirty-eight of the populations' ratios were significantly biased to females (*p*<0.05, binomial test with Bonferroni correction), and no males were found at all in 27 of the populations, i.e. fully unisexual populations. Male individuals were found at residual levels in 11 populations and of those, the male ratio in six populations reached only a few per cent, i.e. these were strongly female-biased populations. A ratio of 20–35% was found in four of the remaining populations, i.e. they were weakly female-biased populations. Many males were found in 20 of the populations, i.e. bisexual populations.

### 3.2 Phylogenetic and phylogeographic analyses

From the results of 16S rRNA haplotype analysis (373 bp) (electronic supplementary material, table S2), 27 haplotypes were observed within the fourteen bisexual populations (from 164 examined specimens), while only one haplotype (S1 haplotype) was observed within the 17 unisexual populations (147 specimens). In the seven strongly female-biased populations (107 specimens) and three weakly female-biased populations (23 specimens), the S1 haplotype was observed in almost all individuals (104 specimens from strongly female-biased populations, and 14 specimens from weakly female-biased populations), yet some individuals had the other haplotypes.

Similarly, from the results of COI haplotype analysis (636 bp; electronic supplementary material, table S3), 60 haplotypes were observed within the 16 bisexual populations (153 specimens). Meanwhile, just one haplotype (C1 haplotype) was observed in all 24 unisexual populations (176 specimens). This C1 haplotype was also found in all females (63 specimens) in the seven strongly female-biased populations (70 specimens) and within three males from the Chikuma-gawa, Ibo-gawa and Ota-gawa strongly female-biased populations. Males had a variety of haplotypes (C2, C30, C31 and C57 haplotypes) in the Natori-gawa (three specimens) and Ota-gawa strongly female-biased populations (one specimen). Although some female individuals had the C1 haplotype in the Ara-kawa and Kando-gawa weakly female-biased populations, all females in the Kizu-gawa and Maruyama-gawa weakly female-biased populations had other haplotypes.

From BI trees based on the sequence data of the 16S rRNA region (electronic supplementary material, figure S2), although monophyly of all of the Japanese *E. shigae* populations was strongly supported by BPP=100, and included the S1 haplotype, phylogeny within the clade was not resolved. According to BI trees based on COI sequences (electronic supplementary material, figure S3), the monophyly of Japanese populations was also fully supported (BPP=100). The clade could be subdivided into four major subclades with high confidence (BPP≧95) and subclade II with low confidence (BPP=87); i.e. subclade I from the eastern Japanese populations, excluding the C1 haplotype. The other subclades II–V were from the western Japanese populations. The monophyly of subclades III–V was strongly supported. However, for subclades II–V, and subclades I and II, it was not. It was concluded that subclade V contained the C1 haplotype, as was observed in all unisexual populations.

**Figure 2.**
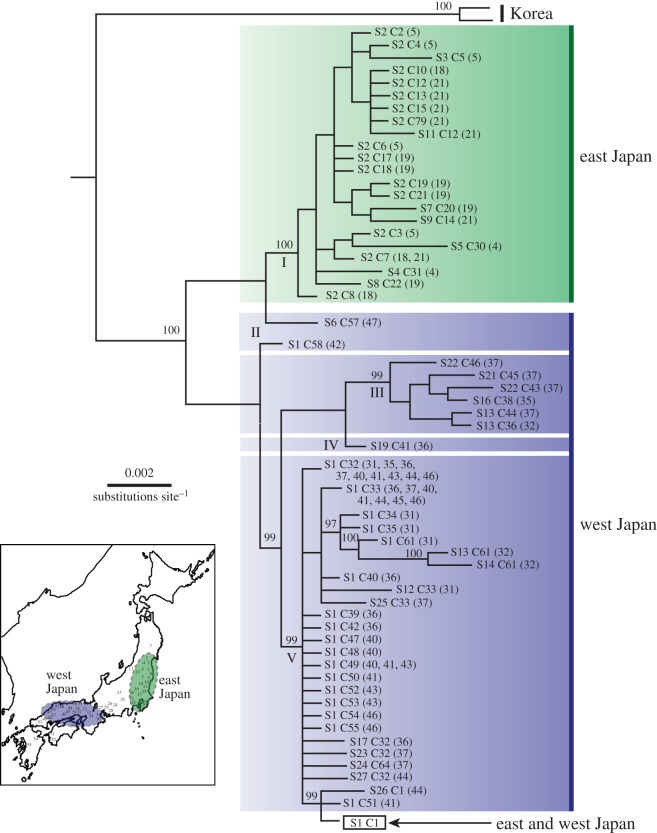
A Bayesian analysis tree of *Ephoron shigae* based on 16S rRNA (373 bp)+COI (636 bp). Operational taxonomic units (OTUs) are indicated by sequence and population numbers (in parentheses) (figure 1; electronic supplementary material, tables S1 and S4). All specimens from unisexual populations across Japan were found to possess the same respective sequence types in each gene region (the ‘S1 C1’); BPP≧94 is indicated above the branches. Roman numerals under the branches indicate major subclades. ESS values are more than 515 for all parameters.

The BI tree based on both 16S rRNA and COI sequences (figure 2; electronic supplementary material, table S4) was almost identical to the BI tree based on just the COI region except subclade II, and the monophyly of Japanese populations of each subclade (I, III and V) was fully supported (BPP≧99). The monophyly of subclade I from eastern Japanese populations excluding the S1–C1 haplotype-set, subclade II and subclades III–V from western Japanese populations was highly supported (BPP≧99). Subclade II formed an unreliable clade with either subclade I or subclades III–V. Subclade V was concluded to be the S1–C1 haplotype-set, as was observed in all unisexual populations.

**Figure 3.**
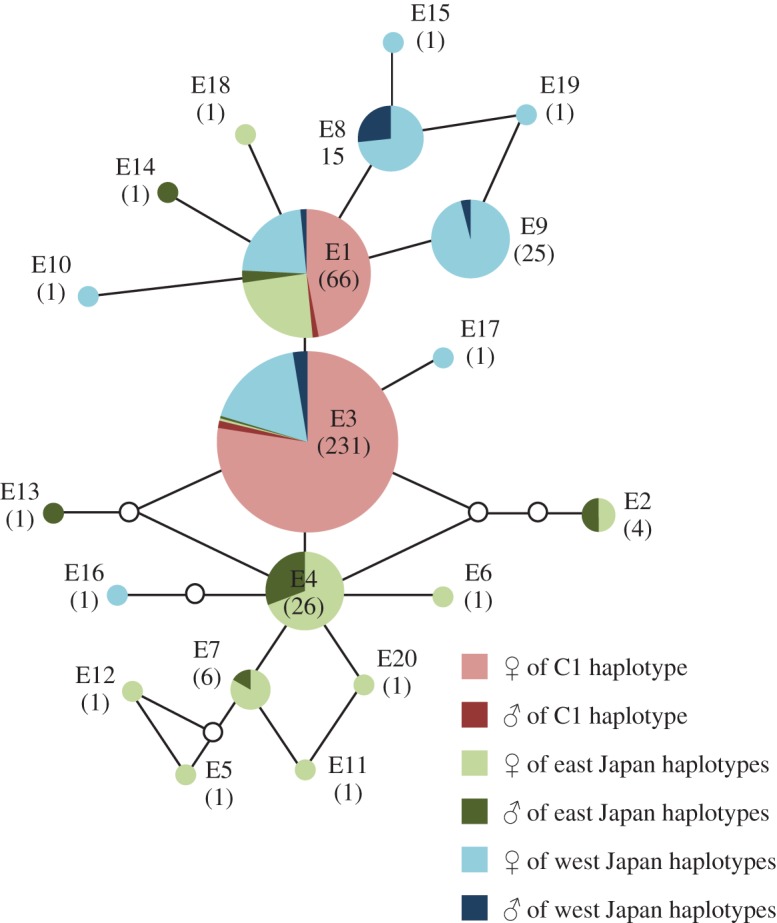
Statistical parsimony network for the nuclear EFI-*α* sequences of 618 base pairs. Solid branches connect sequence types with a single step (missing intermediates are indicated by an open circle). Each sequence type is defined in the electronic supplementary material, table S5 and divided into class by sex and COI haplotype analysis (electronic supplementary material, table S3 and figure S2). Individual numbers are indicated in parentheses. Heavy lines encompass phylogroups where the sequence types are connected with 95% probability. All specimens from unisexual populations across Japan were found to possess the same respective sequence types (the ‘E1’ and ‘E3’).

By means of nuclear EFI-*α* sequences (618 bp), 20 sequence types were presumed within the 38 Japanese populations; 17 unisexual populations (96 specimens), four strongly female-biased populations (26 specimens), four weakly female-biased populations (44 specimens) and 13 bisexual populations (69 specimens) (figure 3; electronic supplementary material, table S5). The network tree resulted in a star-like shape, with the most frequent haplotypes in the centre of the network tree, surrounded by several lower frequency haplotypes; a pattern typically observed in expanding populations. Females with the C1 haplotype according to the COI region were observed in all the population types. In addition, all females were heterozygous of the E1/E3 sequence genotypes and homozygous of the E1/E1 or E3/E3. We detected the C1 haplotype in males at the mtDNA COI region in Chikuma-gawa and Ibo-gawa populations having a homozygous E3 genotype at this nuclear DNA EFI-*α* region, or having heterozygous E1/E3 genotypes, as well as females in the populations, respectively. Males in other populations had other types besides the E1 and E3 genotypes.

The AMOVA of mitochondrial 16S rRNA and COI genes and nuclear EFI-*α* produced similar results, indicating that a high proportion of overall genetic variation occurred at among-group levels distinctly in each of the eastern versus western Japanese populations (table 1).

**Table 1.** AMOVA of the bisexual populations for mitochondrial 16S rRNA and COI and nuclear EFI-*α* genes. (*p<0.01, ^**^p<0.001.)

source of variation	d.f.	S.S.	variance components	% variation	fixation index
16S rRNA					
among groups (eastern versus western Japan)	1	107.7	1.740	80.49	*Φ*ct=0.805*
among populations within groups	12	18.6	0.110	5.09	*Φ*sc=0.261^**^
within populations	152	47.4	0.312	14.42	*Φ*st=0.856^**^
COI					
among groups (eastern versus western Japan)	1	829.2	11.216	84.73	*Φ*ct=0.847^**^
among populations within groups	14	99.6	0.642	4.85	*Φ*sc=0.318^**^
within populations	137	188.9	1.379	10.42	*Φ*st=0.896^**^
EFI-*α*					
among groups (eastern versus western Japan)	1	441.3	6.392	52.55	*Φ*ct=0.526*
among populations within groups	11	46.8	−0.186	−1.53	*Φ*sc=−0.032
within populations	125	744.6	5.957	48.98	*Φ*st=0.510^**^

### 3.3 Historical demography and divergence times

**Figure 4.**
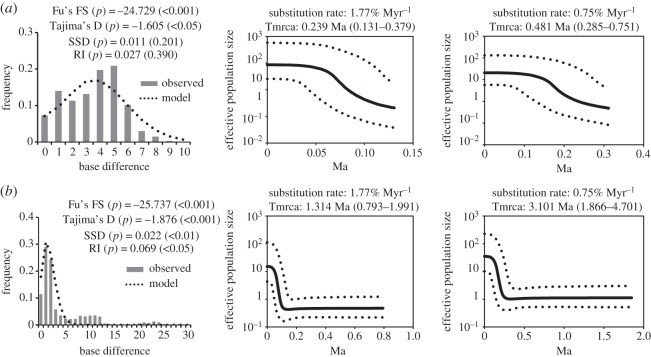
Mismatch distributions of the pairwise nucleotide divergence and Bayesian skyline plot of eastern (*a*; subclade-I of figure 2; electronic supplementary material, figure S3) and western (*b*; subclades II–V) Japanese populations based on the COI gene. Mismatch distributions exclude unisexual populations and individuals with the parthenogenetic strain-specific C1 haplotype in strongly and weakly female-biased populations. Bayesian skyline plots are based on substitution rates of 1.77% divergence lineages^−1^ Myr^−1^ and 0.75%. Tmrcas show median and 95% HPD of lower and upper in parentheses. ESS values are more than 260 for all parameters.

The values of Fu's FS and Tajima's D for eastern populations excluding the C1 haplotype (figure 4*a*) and western populations excluding unisexual populations (figure 4*b*) were negative and significantly different from 0. Therefore, Japanese *E. shigae* has probably experienced periods of population expansion and past population growth. Eastern Japanese populations were also confirmed to have had such periods by inference of the observed mismatched distributions, which closely followed the typical pattern expected of populations that have undergone sudden expansion phases. However, this model was not supported in western Japanese populations. Western Japanese populations exhibited multimodal mismatch distribution patterns, indicating a relatively stable population throughout time.

Bayesian skyline plot analyses were conducted separately using haplotypes found in the eastern (subclade I; figure 2; electronic supplementary material, figure S3) and western Japanese lineages (subclades II–V; figure 4). The Bayesian skyline plot for eastern and western Japanese groups with a 95% highest posterior density (HPD) demonstrated that populations had undergone a major and sudden population growth phase commencing approximately 0.12 Ma (1.77% divergence lineages^−1^Myr^−1^) and 0.32 (0.75%). After this period, the population grew rapidly with a *ca* 35-fold increase by the end of the Last glacial period around 0.012 Ma, and no subsequent decline was observed.

The Tmrcas based upon the sequence data of the COI region for eastern Japanese lineages were estimated to be 0.24 Ma (95% HPD=0.131–0.379 Myr) and 0.48 (0.285–0.751) by the substitution rates, 1.77 and 0.75% Myr^−1^, respectively (figure 4*a*). Western Japanese lineages were 1.31 Ma (0.793–1.991) and 3.10 (1.866–4.701) (figure 4*b*). In addition, the Tmrca for subclade V containing the C1 haplotype (figure 2; electronic supplementary material figure S3) was estimated to be 0.20 Ma (0.121–0.319) and 0.48 (0.285–0.751).

## 4. Discussion

### 4.1 Population history and the dispersion of monophyletically parthenogenetic strains

The results of AMOVA and Mantel testing indicated that geographical distance has primarily restricted gene flow among bisexual populations of *E. shigae*. This is owing to the dispersal ability of this mayfly being extremely weak. In particular, the degree of genetic differentiation between the eastern and western Japanese populations was extremely large, as already reported in our previous study [12]. With respect to the relationship between their pairwise genetic and geographical distances, gene flow between eastern and western Japanese populations is predominantly prevented by physical barriers such as the Japan Alps and Mount Fuji, which are the highest mountains in Japan that formed during the Pliocene [41,42].

The phylogenetic analysis by 16S rRNA and COI of mitochondrial DNA clarified that the unisexual populations were certainly monophyletic in origin rather than polyphyletic, and the unisexual strains recently differentiated somewhere within western Japan. In many other unisexually reproductive organisms, it is generally reported that the parthenogenetic system operates by means of clonal breeding of multiple parallel origins from some bisexual reproductive strains of genetically variable ancestors [4,11,43,44]. Although several exceptions have been observed, such as bdelloid rotifers [45], in the case of parthenogenetic populations scattered in multiple localities, parthenogenetic lineages are mostly polyphyletic in animals [4]. Recent studies addressing insect parthenogenesis also showed multiple origins of parthenogenetic strains [46–48]. Therefore, we can say that geographical parthenogenesis of *E. shigae* is a very rare instance of monophyly.

However, these parthenogenetic strains of *E. shigae* were of relatively recent origin. In this regard, it is very different from the case of the bdelloid rotifers which are of a singular monophyletic origin, similar to this mayfly, yet they are thought to have differentiated millions of years earlier [45].

Both the Tajima's D and Fu's FS analyses for *E. shigae* indicate a relatively recent population expansion. In addition, the population size of this mayfly has grown during the glacial cycles, according to our skyline plot analysis based on the standard substitution rate per Myr for insect mitochondrial DNA sequence data (1.77%; [37] and 0.75%; [38,39]). These parthenogenetic strains of *E. shigae* may also have originated in that age.

It is known that parthenogenetic ability is efficient during population reduction as it would be during a glacial period and the recolonizing of new populations [49,50]. Obligatorily parthenogenetic reproduction resulted in a relatively high dispersal and colonization ability, so unisexual populations of parthenogenetic strains have come to be more widely distributed than those of a bisexual lineage, i.e. eastern and western Japanese bisexual lineages have come to be excluded by parthenogenetic strains.

### 4.2 The reproductive mode of parthenogenetic strains

All of the parthenogenetic females had the C1–S1 haplotype-set in the mitochondrial DNA COI and 16S rRNA regions, while in the nuclear DNA EFI-*α* region, it was revealed that they have the heterozygous genotype of E1/E3, and the homozygous genotypes of E1/E1 or E3/E3. These instances of obligatory parthenogenesis are automictic in type, that is to say, the female prenucleus fuses into the second polar body following the completion of meiosis [18]. Such automictic parthenogenesis is generally understood to lead to a decrease in heterozygosity over a number of generations [1,2,11,51,52], and this was found to be consistent with the results of this present study. The ancestors of the parthenogenetic strains were probably heterozygous, and of the E1 and E3 types. Since the time of their existence, automictic parthenogenesis caused a gradual decrease in the degree of heterozygosity.

In the geographically parthenogenetic ostracods, *Eucypris virens*, the disagreement between the nuclear and mitochondrial DNA data verified the fact that genetic exchange has taken place between the bisexual and unisexual lineages [53]. In *E. shigae*, in which unisexual populations are scattered amongst the bisexual populations throughout Japan, the nuclear DNA data corresponded to that of the mitochondrial DNA data. Therefore, we suggest that female individuals of the parthenogenetic strains came to lack the ability to mate with males, and so oviposit any fertilized eggs.

In the sex ratio study, female-biases were found in some populations, i.e. including both strongly and weakly female-biased populations. All female individuals of the Natori-gawa strongly female-biased population possessed the specific S1 (the 16S rRNA region) and C1 (the COI region) mitochondrial haplotypes. However, in the Ara-kawa and Kando-gawa weakly female-biased populations, many female and male individuals possessed different haplotypes from the specific haplotype in all of the unisexual populations. Therefore, the parthenogenetic strains have probably mingled into what were probably originally bisexual populations. We suggest that initial instances of invasion and subsequent increases in the dominance of the parthenogenetic strains which lack bisexual ability gradually caused a bias towards more female-dominated bisexual populations.

In this study, the extremely low number of males of the Chikuma-gawa, Ibo-gawa and Ota-gawa strongly female-biased populations possessed the unisexual population-specific haplotypes. Because parthenogenetic strains have become differentiated from bisexual ancestral lineages possessing the S1 and C1 haplotypes, it is certain that males having the S1 and C1 haplotypes existed in the western Japanese populations. However, it would have been difficult for the Chikuma-gawa population to have maintained males by fertilized eggs of a parthenogenetic strain, because in the Chikuma-gawa population, no male has been observed even with a lot of sampling over a long period (approx. 30 years) [54]. In some other populations (e.g. the Shonai-gawa, Hiji-gawa and Oita-gawa Rivers), it was similarly reported in previous studies that male individuals were rarely observed (males<1%) [15,16]. At the time of the present study, we were unable to collect a single male within these populations. Because no bisexual populations were found around these populations, it is hard to conceive that any rare males ever found within these populations could have been transferred/dispersed from surrounding bisexual populations. Conceivably, these males were generated by some sort of accident within each parthenogenetic population.

Although thelytoky is generally observed in parthenogenetic mayflies (Ameletidae, Baetidae, Baetiscidae, Caenidae, Ephemerellidae, Ephemeridae, Heptageniidae, Leptophlebiidae, Palingeniidae, Polymitarcyidae and Siphlonuridae) [55–62], in some parthenogenetic mayflies, deuterotoky (i.e. generating both males and females from unfertilized eggs) has also been proposed [61]. Determination of sex depends on the sex-chromosome type of the XO (male)/XX (female) in *E. shigae*, and parthenogenetic offspring in unisexual populations become diploid by fusion of the female prenucleus and a second polar body [18]. As a result, males could be generated when one X-chromosome is lost from the spindle of the female prenucleus or a second polar body [63].

We are unable to contradict the possibility of gene flow from the parthenogenetic strains to those of bisexual lineages, although it is unclear as to whether or not males produced from parthenogenetic strains are functional in terms of sexual reproduction. We are still unable to completely exclude the possibility of incomplete isolation between the strains of parthenogenetic and bisexual lineages (cf. [64]). It is important to establish in future studies the structure of the scattered geographical distributions of unisexual populations amongst the bisexual populations in order to confirm whether or not females of parthenogenetic strains are actually able to mate with males and thereafter also lay fertilized eggs.

## Supplementary Material

Table S1 Localities and sex ratios of Ephoron shigae populations

## Supplementary Material

Table S2 16S rRNA haplotypes of Ephoron shigae

## Supplementary Material

Table S3 COI haplotypes of Ephoron shigae

## Supplementary Material

Table S4 Haplotypes of Ephoron shigae based on 16S rRNA + COI

## Supplementary Material

Table S5 EFI-a sequence types of Ephoron shigae

## Supplementary Material

Figure S1 Distribution of E. shigae in Japan. Blue open circles indicate bisexual populations, red solid circles unisexual, purple open stars weakly female-biased, and orange solid stars strongly female-biased. Population numbers are referred to in table S1 (Supporting information).

## Supplementary Material

Figure S2 A Bayesian analysis tree of Ephoron shigae based on the 16S rRNA gene (373 bp). Operational taxonomic units (OTUs) are indicated by sequence and population numbers (in parentheses)(figure 1, table S1 and S2). All specimens from parthenogenetic unisexual populations across Japan were found to possess the same respective sequence types in each gene region (the “S1 haplotype”). Bayesian posterior probability (BPP ≧ 94) is indicated above the branches.

## Supplementary Material

Figure S3 A Bayesian analysis tree of Ephoron shigae based on the COI gene (636 bp). Operational taxonomic units (OTUs) are indicated by sequence and population numbers (in parentheses)(figure 1, table S1 and S3). All specimens from parthenogenetic unisexual populations across Japan were found to possess the same respective sequence types in each gene region (the “C1”). Bayesian posterior probability (BPP ≧ 94) is indicated above the branches. Roman numerals under the branches indicate major subclades.

## Data accessibility

The datasets of localities and haplotypes supporting this article have been uploaded as the electronic supplementary material. DNA sequences: GenBank accessions AB874274–AB874406, AB711583, AB711590, AB711757 and AB711764. Phylogenetic data: TreeBASE accession number S17610. http://purl.org/phylo/treebase/phylows/study/TB2:S17610.
